# A Review of Recent Progress in Drug Doping and Gene Doping Control Analysis

**DOI:** 10.3390/molecules28145483

**Published:** 2023-07-18

**Authors:** Yuze Lu, Jiayu Yan, Gaozhi Ou, Li Fu

**Affiliations:** 1 Laboratory of Biochemistry, School of Physical Education, China University of Geosciences, Wuhan 430074, China; luyuze2001@foxmail.com (Y.L.); yjy@apm.ac.cn (J.Y.); 2 Key Laboratory of Novel Materials for Sensor of Zhejiang Province, College of Materials and Environmental Engineering, Hangzhou Dianzi University, Hangzhou 310018, China

**Keywords:** progress, drug doping, gene doping, doping control analysis

## Abstract

The illicit utilization of performance-enhancing substances, commonly referred to as doping, not only infringes upon the principles of fair competition within athletic pursuits but also poses significant health hazards to athletes. Doping control analysis has emerged as a conventional approach to ensuring equity and integrity in sports. Over the past few decades, extensive advancements have been made in doping control analysis methods, catering to the escalating need for qualitative and quantitative analysis of numerous banned substances exhibiting diverse chemical and biological characteristics. Progress in science, technology, and instrumentation has facilitated the proliferation of varied techniques for detecting doping. In this comprehensive review, we present a succinct overview of recent research developments within the last ten years pertaining to these doping detection methodologies. We undertake a comparative analysis, evaluating the merits and limitations of each technique, and offer insights into the prospective future advancements in doping detection methods. It is noteworthy that the continual design and synthesis of novel synthetic doping agents have compelled researchers to constantly refine and innovate doping detection methods in order to address the ever-expanding range of covertly employed doping agents. Overall, we remain in a passive position for doping detection and are always on the road to doping control.

## 1. Introduction

Doping, which originally occurred when drug stimulants were used to improve the performance of athletes, has evolved into a comprehensive term encompassing prohibited substances [1,2,3]. It is widely recognized that doping poses substantial physical and psychological risks to athletes. Furthermore, the use of doping in competitive sports is a violation of both sporting ethics and fair play, constituting a breach of athletes’ oaths and a sacrilegious contravention of the Olympic Charter. Consequently, the detection of doping remains a pivotal aspect of anti-doping endeavors [4]. However, there are still a number of problems with doping control, including the following: (1) Technical difficulties: Current doping control techniques do not yet fully cover all prohibited substances, and there are also high rates of false detections and missed detections. (2) Detection period: Many doping substances have a short life cycle, and it is a challenge to effectively detect and retain the reliability of field sampling within this short time frame [1].

The principles of existing doping analysis can be summarized as follows: (1) Detection methods: Doping control laboratories use a variety of analytical techniques to detect banned substances in athletes’ samples, including mass spectrometry (MS), gas chromatography (GC), and liquid chromatography (LC). These methods are applied to blood, urine, or other biological matrices, depending on the nature of the substance and its rate of clearance from an athlete’s body. (2) List of prohibited substances: The World Anti-Doping Agency (WADA) maintains a list of prohibited substances and methods that is regularly updated based on scientific research and evaluation. This list includes different categories of substances, such as anabolic agents, peptide hormones, stimulants, narcotics, and others. (3) Athlete testing: Athletes are subject to both in-competition and out-of-competition testing, which can be performed either randomly or based on intelligence-led information. Testing can be conducted through urine, blood, or saliva samples, and it is usually carried out by trained specialists who follow strict protocols to ensure the integrity and accuracy of the results. (4) Consequences of doping: If an athlete tests positive for a banned substance, he or she may face sanctions such as disqualification, suspension, or even expulsion from sports competitions. Additionally, athletes may suffer significant reputational damage and loss of career opportunities due to the negative publicity of being found guilty of doping [2,3,5,6]. In conclusion, the principles of existing doping analysis aim to preserve the fairness and integrity of sports by detecting and preventing the use of banned substances and methods among athletes. These principles rely on sophisticated analytical techniques, a comprehensive list of prohibited substances, and regular athlete testing programs to identify and sanction dopers appropriately.

Nowadays, doping control analysis has become a conventional means to ensure the fairness and justice of sports [3,7]. Traditional doping detection methods mainly rely on two chromatography methods [8]. One is the GC-MS method. And another is the LC-MS method. These two screening methods are complementary, and together they cover all low- to medium-molecular-weight drugs of abuse that need to be detected according to the WADA regulations. For example, some compounds are difficult or impossible to detect by using LC-MS (e.g., low ionization efficiency for oxymesterone), and these need to be included in the GC-MS method [7]. However, with the continuous development of detection technology, new doping detection methods, such as various fluorescence [9], colorimetric [10], electrochemical [11], and biosensors [12], have been used to detect doping. In addition, PCR technology has also been applied in the doping detection field in current emerging doping methods [13]. Through qualitative or quantitative analysis to determine whether there are prohibited substances or metabolites in biological samples (e.g., urine or blood) collected in and out of competition, an anti-doping agency can judge whether athletes use doping or not [14,15,16,17].

According to the different ways in which prohibited substances can enter athletes, the current types of doping can be mainly divided into two categories: drug doping [11,18] and gene doping [13,19]. Drug doping is a general term for organic drugs that are explicitly prohibited by the WADA [1]. Based on whether it is during the competition and some given sports, prohibited substances and methods can roughly be divided into the following three categories: substances and methods prohibited at all times (S0–S5, M1–M3); substances and methods prohibited in competition (S6–S9); and substances prohibited in particular sports (P1) (Table 1).

With the continuous progress of science and technology, genetic modification technologies are becoming increasingly mature. As a result, doping types and methods have become more complex, including the most recent type, gene doping [20]. Gene doping refers to substances or techniques that import substances such as foreign genes or cells into athletes for nontherapeutic purposes to improve athletic performance by improper methods [19,21,22]. By introducing relevant genes into the human body to further express relevant proteins such as insulin-like growth factor, vascular endothelial growth factor, and endorphins, the muscle recovery of athletes can be promoted, thereby enhancing athletic performance by illicit means. Gene doping is more difficult to detect than drug doping [19]. This is due to the high degree of homogeneity between the exogenous genes introduced by gene doping and physiological DNA, which is difficult to identify using non-invasive direct detection methods. Indirect assays that attempt to detect gene vectors, even if they are based on the body’s immune response (e.g., to an adenovirus or other vector), often have difficulty distinguishing whether the immune response originates from a natural infection or an artificially introduced virus.

The existence of such a wide variety of prohibited substances (approximately 250 compounds) [23] has created a heavy burden for doping detection [24]. To meet the increasing demand for qualitative and quantitative analysis of hundreds of substances with widely different chemical and biological properties, the requirements for doping control methods continue to increase, and the detection methods have rapidly developed [25,26,27]. The aim of this review is to present an exhaustive and critical overview of the scientific literature that is related to the significant progress in analytical anti-doping methodologies in the past decade. The advantages and limitations of each analysis method were compared, and the possible development direction of future doping detection methods was also proposed.

## 2. Drug Doping Detection

Detection of drug doping has been a critical part of doping control over the past decades [28]. The development of modern analytical instruments has greatly improved the capability to detect doping agents. In addition to various chromatography and MS-based separation analysis methods, a large variety of fluorescence, electrochemical, and colorimetric methods and many biosensors have shown their powerful capabilities for doping control analysis. However, as novel drug molecules with doping effects continue to be designed, doping control laboratories are urged to develop and refine the relevant testing methods and strategies to provide the WADA with as much information as possible to support anti-doping decisions [29]. Therefore, over the past years, these drug doping detection methods have been constantly updated to enable faster and more accurate determinations of relevant doping drugs.

### 2.1. MS-Based Methods

**Figure 1:**
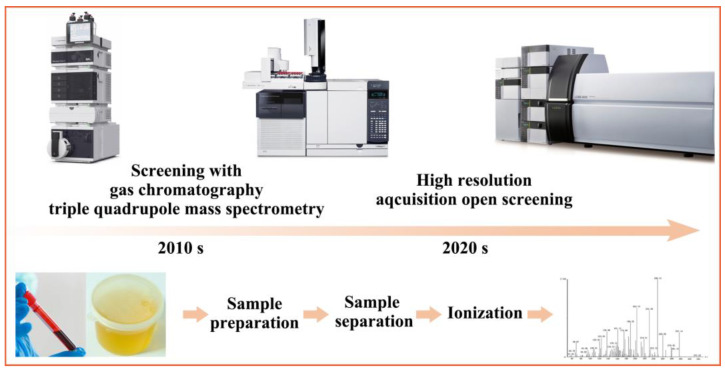
The development history of the MS-based methods for doping control analysis and the specific process of MS for the detection of doping.

Historically, MS-based separation analysis methods have long been the gold standard for doping detection because of their high accuracy, sensitivity, speed, and throughput. As the most classic doping detection methods, GC-MS and LC-MS are widely used in doping screening [7,15,18,30,31,32] (Figure 1). These two screening methods work in synergy, allowing them to cover all low- to medium-molecular-weight drugs of abuse that must be detected according to the WADA regulations [7]. For example, triple quadrupole mass-base spectrometry was widely used in the 2010s, which is a powerful analytical technique that has been widely used in the field of doping detection. It offers high sensitivity and selectivity for the analysis of drugs and their metabolites in biological samples. For example, Voclcker’s group achieved rapid detection of anabolic and narcotic doping agents in saliva and urine by means of nanostructured silicon-based surface-assisted laser desorption/ionization MS. The constructed assay has the capacity for high-throughput analysis of hundreds of biological samples, which provides opportunities for real-time MS analysis at sporting events [8]. It is notable that MS- and MS-based chromatographic techniques provide detailed structural information and enable qualitative as well as quantitative analysis at the trace level for a wide variety of sample types [33,34]. In recent years, with the development of micro/nanofluidics, MS instruments have gradually developed towards integration and miniaturization. Coppieters’ group has developed a nanoflow LC-MS device that enables automated filtration and detection of doping-relevant small peptide hormones in urine samples [35]. This work uses nano-liquid chromatography coupled with electrospray ionization MS (ESI-MS), which not only reduces the amount of sample required for the measurement process but also facilitates lower detection limits by converting the analytical-scale LC instruments to micro/nano LC scale systems. And with the progress of science and technology, MS methods have gradually developed from single MS molecules to dual MS coupled liquid chromatography-tandem MS (LC-MS/MS). This analytical technique combines the separation capabilities of HPLC with the sensitive and selective detection capabilities of MS/MS. In doping detection, HPLC-MS/MS not only offers exceptional sensitivity, allowing for the detection of trace amounts of doping substances, but also provides high selectivity by combining the separation power of HPLC with the specific identification capabilities of MS/MS. For example, Lu’s group has successfully achieved the identification and characterization of higenamine metabolites in human urine by utilizing a LC-MS/MS instrument [5]. Ponzetto’s group has successfully used the UHPLC-MS/MS method for simultaneous quantification of endogenous steroids and their phase II metabolites in serum [36]. The combination of multiplexed mass spectrometers allows for more detailed and accurate analysis of trace doping molecules in complex biological matrices, improving the accuracy and sensitivity of doping detection. And in the 2020s, an ultra-high-performance liquid chromatography-tandem MS (UHPLC-MS/MS) method has been developed for drug doping quantification. UHPLC-MS/MS has revolutionized the field of doping detection due to its high sensitivity, selectivity, and speed. UHPLC-MS/MS brought significant improvements in separation efficiency, peak capacity, and analysis speed. This allowed for the rapid analysis of large numbers of samples, making it ideal for anti-doping laboratories that process a high volume of samples. Lian’s group reports an on-line purification and high throughput platform for fast screening of 39 glucocorticoids in animal-derived foods (pork, chicken, milk, and eggs) using on-line solid-phase extraction technology and liquid chromatography-tandem high-resolution MS (LC-HRMS) [37]. Ai’s group developed a rapid, sensitive, and confirmatory method and validated it for the determination of 14 diuretics in multiple animal-derived foods using UHPLC-MS/MS [6]. In this way, the high throughput technique not only allows for a high analytical efficiency of the MS method but also enables a more comprehensive analysis of the types of stimulants that may be present in the sample to be tested, ensuring the accuracy of the results.

However, beyond the appropriate selectivity, sensitivity, and stability of MS-based methods, a series of shortcomings of the MS method, such as expensive instruments, complex operation, high technical requirements, and complicated sample preparation, should not be ignored [38,39]. In addition, as the conformation of the target doping molecule tends to be hidden and the sample matrix becomes more complex, it is important to develop detection systems with high resolution performance. However, up to now, high resolution technology in combination with LC/GC has not yet been used as a complete LC-MS/GC-MS screening method for doping control analyses. The available LC/GC high-resolution instruments were not sensitive enough for a number of compounds and/or had inadequate linearity to comply with all the required WADA criteria, meaning that the current MS-based screening methods could not be replaced by an equivalent method on GC high-resolution MS [7]. These drawbacks make it particularly important to develop other new doping detection methods, especially in developing countries. And the development of LC-MS or GC-MS methods that are sensitive enough (especially for the exogenous compounds) and as cost-effective as feasible is the way forward for the subsequent development of MS-based methods for drug doping detection [7].

The future development direction of MS in doping detection includes the following aspects: (1) Sensitivity and selectivity improvement: MS techniques will continue to enhance their sensitivity and selectivity for doping detection. This involves improving the detection limits and reducing false-positive and false-negative results. (2) Integration with sample preparation methods: Efforts will be made to integrate MS with efficient sample preparation methods. This can reduce the time and complexity of sample preparation, making the doping detection process more streamlined and effective. (3) Miniaturization and portability: Miniaturized and portable MS will be developed to enable on-site and real-time doping testing. This will benefit sports competitions as it allows for immediate detection and intervention in cases of doping. (4) Method advancements in metabolite and biomarker analysis: The focus will be on developing methods for the analysis of metabolites and biomarkers associated with doping. This will help in the identification of new doping agents and improve the accuracy of detection. (5) Non-targeted analysis: Non-targeted analysis using MS will be further explored. This approach allows for the simultaneous detection of a wide range of compounds, including known and unknown doping substances, providing a comprehensive view of doping in athletes. (6) Advanced data processing and interpretation: The development of advanced data processing and interpretation methods will be crucial in improving the reliability and efficiency of doping detection using MS. This includes the use of artificial intelligence and machine learning algorithms to analyze complex data sets. These future directions aim to continuously improve the capabilities of MS in doping detection, ensuring fair and clean sports competitions.

### 2.2. Fluorescence Methods

**Figure 2:**
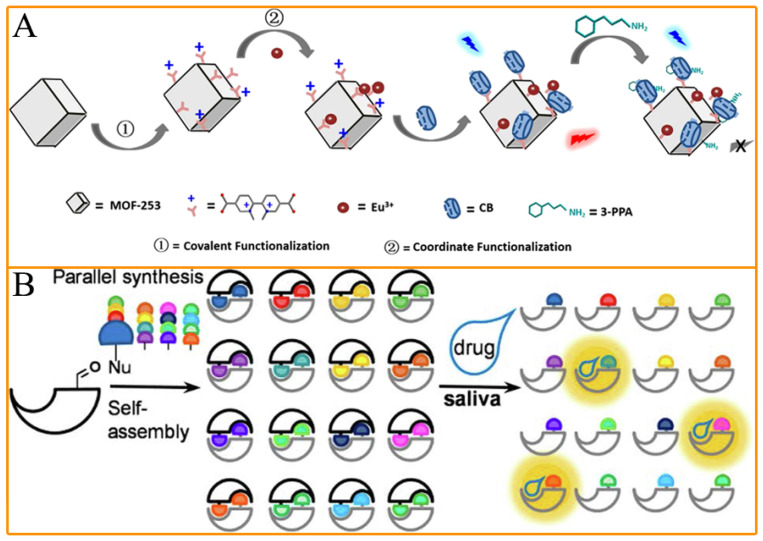
Examples for “signal-on” and “signal-off” fluorescence sensors for doping detection: (**A**) Functional MOF material-mediated host-guest interactions recognize the stimulant 3-phenylpropylamine as a “signal-off” type fluorescent sensor; (**B**) Supramolecular probe-mediated “signal-on” fluorescent sensor for simultaneous detection of multiple types of stimulant drugs in saliva. (Reprinted with permission from [43]. Copyright 2020, Elsevier Publishing (**A**), and [44]. Copyright 2019, American Chemical Society).

Recently, many fluorescent detection platforms have been developed based on the interactions among doping agents and fluorescent probes to generate fluorescent emission complexes. Compared with LC-MS and GC-MS, fluorescence-based methods exhibit high sensitivity, good selectivity, and easy portability [27,40,41]. For example, Cheng’s group used fluorescent covalent polymers (CPs) as the signal carrier to successfully develop a novel fluorescence method to detect drug doping with methamphetamine hydrochloride. The inherent interactions between the target molecules and signal probes led to quantitative fluorescence quenching of CPs to realize the excellent performance of the method [42]. Yan’s research group designed a cucurbit [7] uril-Anchored bis-functionalized metal-organic framework hybrid as the signal probe and used the interactions among the signal probes and amphetamine-type stimulants to cause fluorescence quenching of the signal probes. A signal-off-type fluorescent sensor was successfully constructed to enable the detection of amphetamine-type stimulants [43] (Figure 2A). However, sensors based on this “signal-off” mechanism are easily affected by unknown media or different interferences. Therefore, researchers have also developed many “signal-on” fluorescent sensors for doping detection. For example, Hof’s research group reported a parallel synthesis-driven approach to creating a family of self-assembling dimeric supramolecular chemosensors. The functional unit dimer-dyes enable the sensors to produce fluorescence emissions when detecting micromolar concentrations of a wide range of illicit drugs in water and saliva, thus enabling “signal-on” fluorescence detection of drug doping [44] (Figure 2B). Zhou’s group combined upconverting phosphor technology with a lateral flow assay to achieve point-of-collection detections of morphine and methamphetamine in saliva. The prepared sensor is capable of detecting morphine and methamphetamine in saliva samples with high sensitivity within 15 min. The detection limits of the sensor are 17.5 nM for morphine and 67.0 nM for methamphetamine, which are well below the European Union minimum detection requirements of 70 and 170 nM for morphine and methamphetamine, respectively [9]. Generally, considerable progress has been made in the past decade in developing various fluorescence methods with excellent performance for doping detection.

However, these methods are mainly based on the interactions between the target molecules and the fluorophores, and the relationships between the microstructures of doping agents and such fluorescent substances remain uncertain [45]. Therefore, for some structurally similar doping agents or metabolites, it is difficult to distinguish them using fluorescent sensors. At the same time, the complex composition of the biological matrix places particular demands on the anti-interference performance of the sensors. It is worth mentioning that, up until now, most fluorescence sensing was based on fluorescence quenching caused by photo-induced charge transfer between the flourophore and the analyte, which was easily affected by medium or different interferes. Consequently, it is imperative that a specific fluorescence “turn-on” sensor for detecting drug doping with high sensitivity, high selectivity, and a quick response be developed.

The future development direction of fluorescence detection in doping includes the following aspects: (1) Sensitivity enhancement: Efforts will be made to improve the sensitivity of fluorescence detection methods for doping analysis. This involves developing more sensitive fluorophores and improving detection systems to achieve lower detection limits. (2) Multiplexing capability: Future developments will focus on increasing the multiplexing capability of fluorescence detection techniques in doping analysis. This will enable the simultaneous detection of multiple doping substances or metabolites, providing a more comprehensive and efficient analysis. (3) High-throughput screening: Emphasis will be placed on developing high-throughput screening methods using fluorescence detection. This will enable the rapid analysis of large numbers of samples, which is particularly important for anti-doping agencies during major sporting events. (4) Miniaturization and portability: Advances will be made in miniaturizing fluorescence detection devices, making them more portable and user-friendly. This will allow for on-site doping testing, reducing the time and cost associated with sample transportation to a centralized laboratory. (5) Integration with sample preparation techniques: Integration of efficient sample preparation techniques with fluorescence detection methods will be explored. This will simplify the doping analysis process and enhance the reliability and accuracy of the results. (6) Use of nanotechnology: Nanotechnology will play a significant role in the future development of fluorescence detection in doping analysis. Functionalized nanoparticles can be utilized as selective probes for doping agents, enhancing the sensitivity and specificity of detection. (7) Development of advanced data analysis algorithms: Advanced data analysis algorithms, such as machine learning and pattern recognition, will be employed to analyze complex fluorescence data sets. This will facilitate the identification of doping agents and patterns, improving the efficiency of doping detection. These future directions aim to advance the capabilities of fluorescence detection in doping analysis, contributing to the ongoing efforts to ensure fair and clean sports competitions.

### 2.3. Electroanalytical Methods

**Figure 3:**
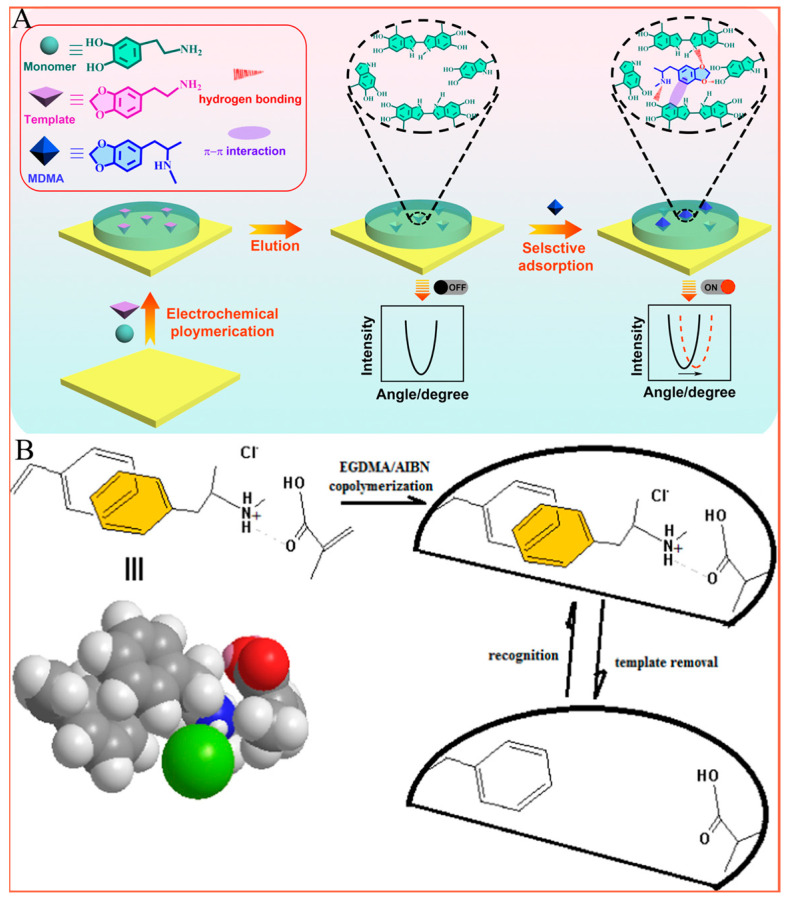
Schematic diagram of (**A**) Electrochemical sensors with molecularly imprinted polymers as recognition units simultaneously detecting two amphetamine-type stimulants; (**B**) π-π stacking between the target molecules and the template molecules as a force for target doping recognition and fast Fourier transform square wave voltammetric determination of methamphetamine stimulant drug. (Reprinted with permission from [50,51]). Copyright 2022 and 2019, Elsevier Publishing).

Similar to fluorescence methods, electroanalytical methods also have high sensitivity. Therefore, they have been widely used in the anti-doping field over the past decade, especially for some doping agents or related metabolites with electrochemical activities [11]. These methods have been gaining prominence and are characterized as simple, rapid, low-cost, and user-convenient with low matrix effects, and there is the possibility of miniaturizing the experimental set-up and using portable electrochemical devices [46]. In addition, electrochemical analytical methods are also considered to be sufficiently sensitive, precise, and accurate, which constantly brings them to the forefront in the field of doping control analysis [47]. According to the different identification methods used in the electrochemical detection process, the electrochemical detection modes of doping agents can roughly be divided into two categories: characteristic redox potential electrochemical sensors [48,49] and molecularly imprinted electrochemical sensors [46,50]. For example, Tarley’s group developed a reliable and selective voltammetric method to detect the designer drug, 1-(3-chlorophenyl) piperazine using a boron-doped diamond electrode. The prepared electrochemical sensors exhibited excellent interference resistance due to the presence of an auxiliary reagent, sodium dodecyl sulfate. Furthermore, the developed method was applied to synthetic samples, and the accuracy was determined by comparison with liquid chromatography with a diode array detector as the reference method [48]. Over the past decade, molecularly imprinted electrochemical sensors have also been extensively developed to further improve the anti-interference capability of electrochemical methods for detection in biological samples. For molecularly imprinted electrochemical sensors, the specific detection of target doping was achieved by means of polymer-mediated molecular imprinting modified on the electrode surface [50]. The molecularly imprinted polymer that can specifically recognize a target molecule can be completed by adding the target doping as an imprinted molecule during the polymer aggregation process and eluting the imprinted molecule after the polymerization is complete [46]. For example, Han’s group developed a novel electrochemical-surface plasmon resonance sensor for amphetamine-type doping detection based on a molecularly imprinted strategy. By using 3,4-methylenedioxyphenethylamine as the template molecule and dopamine as the functional monomer, a molecularly imprinted polymer with specific recognition of amphetamine-type stimulants was successfully loaded on the surface of a surface plasmon resonance chip by means of a one-step electrochemical polymerization method. The prepared sensor showed lower detection limits of 57 nM and 59 nM for 3,4-methylenedioxeamphetamine (MDA) and 3,4-methylenedioxymethamphetamine (MDMA), respectively, with broad linearity. Additionally, the prepared sensor could be resistant to interference from various other illicit drugs and other substances through hydrogen bonding between the target molecule and the template molecule [50]. (Figure 3A). In addition to this, the π-π stacking between the target molecules and the template molecules can also act as a force for target doping recognition. Alizadeh’s group reported an efficient voltammetric method for trace level monitoring of methamphetamine (MTM), a stimulant drug, in human urine and serum samples [51]. This method is based on the fast Fourier transform square wave voltammetric (FFT-SWV) determination of MTM at a molecularly imprinted polymer/multi-walled carbon nanotube modified carbon paste electrode. The fabricated electrochemical sensor exhibits great resistance to interference through π-π stacking interactions between the target molecules and the template molecules. The proposed sensor exhibited a linear response range of 1.0 × 10^−8^–1.0 × 10^−4^ mol L^−1^ and a detection limit of 8.3 × 10^−10^ mol L^−1^ with acceptable relative standard deviations (RSD%) for real samples (1.0–3.5%) (Figure 3B).

Although many electroanalytical methods have shown excellent performance superiority for the simple and sensitive detection of doping agents, it should also be noted that the poor detection stability of electrochemical sensors makes it difficult for them to produce strong and convincing results. In addition, the direct use of characteristic redox peaks as a method to distinguish different types of doping agents lacks the consideration of avoiding interference from coexisting substances in complex biological matrices. Therefore, in modern analytical chemistry, electrochemical analysis is more frequently used as a signal detection tool. By coupling a bio-recognition unit with specific recognition properties, electrochemical detection-based biosensors were constructed. We will discuss this in the section on biosensors. Moreover, it should not be overlooked that electrochemical assays are generally known for their high sensitivity, but they require skilled techniques and can be highly sensitive to measurement conditions. In other words, the preparation of chemically modified electrodes is often time-consuming and more expensive with messy modification protocols, including the complex process of incorporation of a modifier onto the electrode surface, and frequently comprises the synthesis of polymeric matrices and composites [11,46,47]. All these aspects may result in substantially higher background current and unrepeatable results, mostly as a consequence of the non-reproducibility of the preparation of the particular chemically modified substance.

The future development direction of electroanalytical detection in doping can be summarized as follows: (1) Miniaturization and Portability: The trend is to develop smaller and more portable electroanalytical devices for doping detection. This will enable on-site analysis and real-time monitoring, making it easier to detect doping in sports events or in anti-doping control. (2) Sensitivity and Selectivity Improvement: There is a constant need to enhance the sensitivity and selectivity of doping detection methods. Research efforts are being directed towards the development of novel sensing materials, such as nanomaterials, molecularly imprinted polymers, and biomimetic receptors, which can significantly improve the detection limits and reduce false positive/negative results. (3) Multiplexed Detection: The simultaneous detection of multiple doping compounds is becoming increasingly important. Developing electroanalytical methods capable of detecting a wide range of prohibited substances simultaneously will help improve the efficiency and accuracy of doping control. (4) Integration with Other Analytical Techniques: Combining electroanalytical techniques with other analytical methods, such as MS and chromatography, can provide complementary information and enhance the reliability of doping detection. Integrated platforms that combine different techniques are being explored for more comprehensive doping analysis. (5) Automation and High-Throughput Analysis: Automation and high-throughput analysis are crucial for efficient doping testing, especially in large-scale sports events. The development of robotic systems and advanced data processing algorithms will enable faster and more accurate doping detection. (6) Advances in Data Analysis: With the growing complexity of doping substances and the increasing amount of data generated, advanced data analysis techniques, including chemometrics, machine learning, and artificial intelligence, will play a key role in improving the interpretation and reliability of doping test results. (7) Anti-Doping Regulations and Policy: The development of electroanalytical detection methods should be closely aligned with the evolving anti-doping regulations and policies. Continuous collaboration between scientists, sporting authorities, and regulatory agencies is essential to ensuring the effective implementation of electroanalytical techniques in anti-doping efforts. It is important to note that advancements in doping detection techniques should always be guided by ethical considerations and the principles of fair play in sports.

### 2.4. Colorimetric Methods

**Figure 4:**
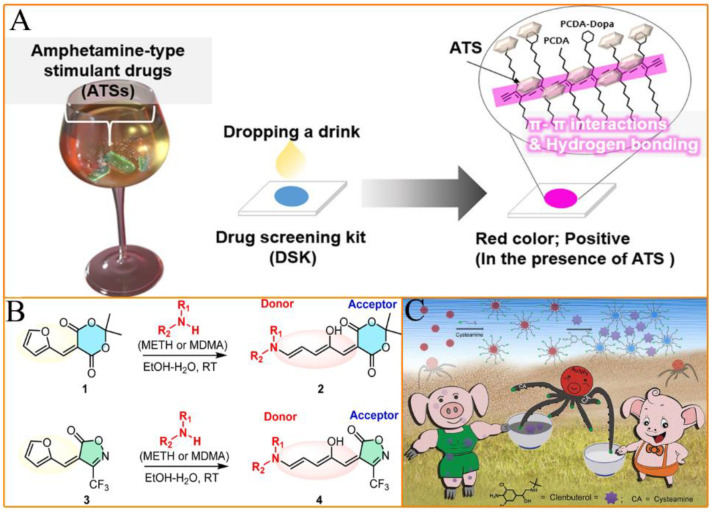
Schematic diagram of (**A**) rapid colorimetric detection of amphetamine-type stimulants via hydrogen bonding and π-π interactions between the drug doping agents and sensing materials; (**B**) sensitive and fast colorimetric detection of amphetamine through the mechanism of donor-receptor (doping and probes) adduct formation; and (**C**) cysteine-modified Au NPs as a signal probe to achieve a rapid colorimetric detection of clenbuterol through the interaction of target molecules and the surface groups of gold nanoparticles. (Reprinted with permission from [26] Copyright 2022, American Chemical Society (**A**), [38] Copyright 2022, Elsevier Publishing (**B**), and [39] Copyright 2016, American Chemical Society).

Compared to electrochemical analysis methods, colorimetric assays may have slightly lower sensitivity but offer the significant advantage of visually detecting targets. This facilitates rapid screening and real-time detection of doping substances in the field. [38]. Recently, colorimetric methods have attracted extensive attention in the doping detection field due to their attractive properties, such as easy operation, real-time, low-cost, and on-site analysis [10,26,38,39]. Simple and rapid visual indications of drug doping can be achieved by using the interaction forces between the stimulants and identification probes to cause color variations in the reaction system. For example, Lim et al. achieved rapid colorimetric detection of amphetamine-type stimulants via hydrogen bonding and π−π interactions between the drug doping agents and sensing materials [26] (Figure 4A). Kim’s group successfully achieved sensitive and fast colorimetric detection of amphetamine through the mechanism of donor-receptor (doping and probe) adduct formation. The activated furan-based probes (compound **1** and compound **3**) can form coloured donor–acceptor Stenhouse adducts (compound **2** and compound **4**) upon binding to the target doping molecule [38] (Figure 4B). Additionally, the unique optical properties of gold nanoparticles (Au NPs) offer possibilities to transduce molecular interactions into detectable colorimetric signals that can be observed by the naked eye. Wu’s group used cysteine-modified Au NPs as a signal probe to achieve rapid colorimetric detection of clenbuterol through the interaction of target molecules and the surface groups of gold nanoparticles [39] (Figure 4C). It is worth mentioning that the sensor prepared based on this work showed good anti-interference performance and can successfully be applied to detect clenbuterol in real blood samples.

It is worth mentioning that all of these colorimetric methods can be further integrated on colorimetric test strips, thus truly enabling instant colorimetric detections in the field. However, it should not be ignored that although colorimetric analysis is widely used in the doping control field, the sensitivity of the method itself makes it difficult to detect low concentrations of doping agents. Poor sensitivity of the colorimetric test when there are low levels of target analogue may be a significant contributing factor to false-negative results. At the same time, certain complex co-existing substances in biological samples may interfere with the detection results of the sensors. More importantly, the handling of color spot reagents also poses an increased health risk as many of the chemicals being used are highly corrosive and toxic. There is therefore an increased interest in the development of highly selective, ultrasensitive, rapid, and safe-to-use color spot tests for accurate on-site testing [10,26,39].

The future development direction of colorimetric detection for doping includes the following aspects: (1) Sensitivity improvement: Efforts will be made to enhance the sensitivity of colorimetric detection methods by employing novel materials and optimizing experimental conditions. This will enable the detection of doping agents at lower concentrations, making the method more reliable and practical. (2) Multiplex detection: Researchers are working towards developing colorimetric detection methods that can simultaneously detect multiple doping agents in a single test. This will provide a more comprehensive analysis and save time and resources compared to traditional methods. (3) Portable and rapid detection devices: There is an increasing demand for on-site and real-time detection of doping agents, especially in sports competitions. Future development will focus on creating portable and rapid detection devices that are easy to operate, sensitive, and deliver results quickly. (4) Integration of emerging technologies: Colorimetric detection methods can benefit from the integration of emerging technologies such as nanomaterials, microfluidics, and biosensors. These technologies have the potential to improve the accuracy, sensitivity, and reliability of colorimetric doping detection. (5) Standardization and validation: To ensure the widespread adoption of colorimetric detection methods for doping, it is crucial to establish standardized protocols and validate the performance of these methods. This will enable reliable and consistent results across different laboratories and facilitate the acceptance of colorimetric detection in both research and practical applications.

### 2.5. Biosensors

**Figure 5:**
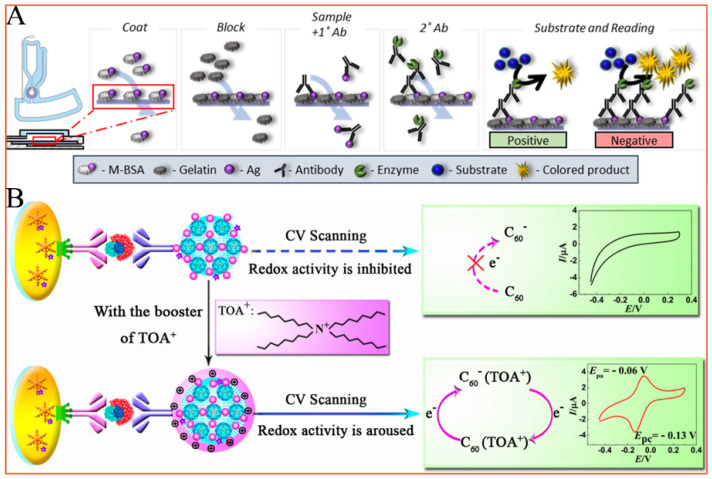
Schematic diagram of (**A**) the specific recognition effect of the biological antibodies and semiquantitative detection of morphine with the help of a centrifugal microfluidic colorimetric enzyme-linked immunosorbent assay; (**B**) the hydrophilic C60-based nanomaterial and the constructed sandwich-type immunosensor for erythropoietin detection based on the inner redox activity of fullerene. (Reprinted with permission from [4,14] Copyright 2021 and 2015, American Chemical Society).

Biosensors are a category of useful analytical devices that are constructed using combinations of different bioactive materials and physical/chemical signal transducers. Because of their unique advantages, such as high sensitivity, simple operation, good selectivity, and low sample consumption, biosensors can be easily applied to accurately and rapidly detect a large variety of analytes, including various doping substances in complex systems. Conventional biosensors are commonly constructed based on highly specific antibody-antigen immunoreaction reactions. Based on the immobilization of the corresponding antibodies on certain substrates to capture antigen molecules and the utilization of proper optical or electrochemical methods to output and amplify the weak immunorecognition information, many immunosensors have played indispensable roles in the field of doping detection [3,12,52]. For example, Dignan’s group used the specific recognition effect of the biological antibodies and achieved precise, cost-efficient, and semiquantitative detection of morphine with the help of a centrifugal microfluidic colorimetric enzyme-linked immunosorbent assay [14] (Figure 5A). Yuan et al. successfully synthesized a hydrophilic C_60_-based nanomaterial and constructed a sandwich-type immunosensor for erythropoietin detection based on the inner redox activity of fullerene (Figure 5B). The proposed immunosensor shows a wide linear range and a relatively low detection limit for erythropoietin [4]. However, it should be noted that the poor biostability of biological antibodies and the high cost of antibody preparation have hampered their large-scale application. Moreover, the problem with immunoassays is that there is a great probability of obtaining a false negative or false positive result due to the ambiguity of detection (in the form of faint stripes), degradation of the antibodies used, and cross-reactivity with other analytes. Some studies performed with various commercially available assays revealed a 70% false positive and sometimes 50% false negative detection accuracy [53]. Thus, immunoassays are used as preliminary screening approaches in situ, which are then followed by a chromatographic technique to confirm the results.

**Figure 6:**
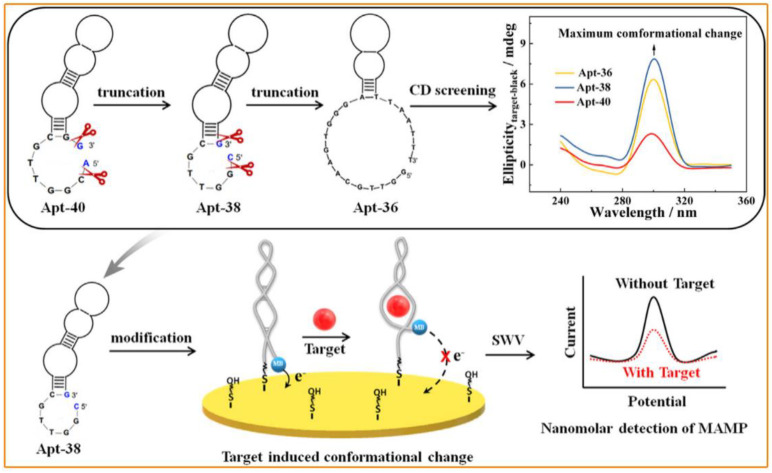
Schematic diagram of the aptamer sequence of methamphetamine as the recognition unit to achieve the electrochemical detection of methamphetamine by square wave voltammetry. (Reprinted with permission from [16] Copyright 2022, Elsevier Publishing).

Interestingly, the wide screening of aptamers through the in vitro SELEX technique for bioassay applications has resulted in a broad development prospect for improving biosensor performance [54]. Compared with immunosensors, aptamer-based biosensors have lower detection costs, higher biostability, and better interference resistance and are widely used in the field of doping detection. Therefore, they can serve as versatile biorecognition elements for specifically binding a wide variety of target molecules. Undoubtedly, doping agents can also be used as target molecules. Over a period of time, biosensors constructed using aptamers as recognition units have been extensively used in the field of doping detection [16,55]. For example, Sun’s group used the aptamer sequence of methamphetamine as the recognition unit to achieve the electrochemical detection of methamphetamine by square wave voltammetry (Figure 6). The constructed sensor exhibited good detection sensitivity. The detection limit was far below the clinical detection threshold, which is very conducive to large-scale promotion and application [16]. Not surprisingly, due to the unique advantages of aptamers in target recognition [56], biosensors have the potential to become the main method used for doping detection in the future. Much progress has been made regarding immune-based sensors. However, to further enrich the application of immunosensors in the field of doping detection, constructing a high-throughput immunoassay that can achieve simultaneous detections of multiple types of doping agents is an important direction for future development. In addition, biochips are also an important development direction for doping detection. Therefore, the future development of biosensors based on immune recognition and aptamer recognition will continue to focus on the development of more rapid and sensitive biosensors with higher detection throughput.

The future development direction of biosensors for doping detection may include the following aspects: (1) Improved Sensitivity: Enhancing the sensitivity of biosensors will enable the detection of even lower concentrations of doping substances in biological samples, improving the accuracy and reliability of the detection results. (2) Multianalyte Detection: Developing biosensors capable of simultaneously detecting multiple doping substances will be crucial in combating the use of various banned substances by athletes. This can be achieved by integrating different types of recognition elements or utilizing advanced nanotechnology. (3) Miniaturization and Portability: Miniaturizing biosensor devices will allow for on-site and real-time monitoring of doping, making it more convenient and accessible for authorities to carry out tests during sporting events. Portable biosensors will also facilitate the detection of doping in athletes’ training environments. (4) Non-Invasive Detection: Advancements in non-invasive sampling techniques, such as saliva or sweat-based detection, can minimize the discomfort experienced by athletes during sample collection. Biosensors that can detect doping substances from these alternative sample sources may gain popularity in the future. (5) Enhancing Selectivity: Improving the selectivity of biosensors will help reduce false-positive and false-negative results in doping detection. This can be achieved through the development of specific recognition elements or the integration of advanced signal processing algorithms. (6) Integration with Data Analysis: Incorporating biosensors with data analysis tools, such as machine learning algorithms, can enhance the interpretation of detection results. This integration can provide valuable insights into patterns, trends, and individual athlete profiles related to doping. (7) Increased Affordability: Making biosensor technology more affordable will promote wider adoption and accessibility, not only in professional sports but also in grassroots and amateur-level competitions. Cost reduction can be achieved through advancements in manufacturing processes and scalable production methods. It is important to note that the future development of biosensors for doping detection will also require continuous collaboration between researchers, sports authorities, and regulatory bodies to address emerging doping techniques and stay ahead of the challenges posed by the advancement of doping substances.

## 3. Gene Doping Detection

Gene doping refers to the use of genetic engineering techniques to enhance athletic performance. There are several methods and types used in gene doping, each with its own advantages and disadvantages. (1) Gene therapy: This involves the introduction of a healthy copy of a gene into a person’s cells to correct a genetic defect or enhance a particular trait. This can be conducted using a virus that has been modified to carry the desired gene. Gene therapy has been used in humans to treat certain genetic disorders, such as cystic fibrosis and muscular dystrophy. (2) Epigenetic modification: This involves altering the way genes are expressed without changing the actual DNA sequence. For example, by modifying the chemical tags (such as methyl groups) attached to the DNA, it is possible to turn genes on or off, which can affect athletic performance. (3) Gene editing: This involves using molecular tools such as CRISPR/Cas9 to precisely edit the DNA sequence of an organism. Gene editing could be used to enhance athletic performance by introducing beneficial mutations or removing harmful ones. (4) Gene transfer: This involves the transfer of genes from one organism to another. For example, by transferring genes for increased muscle growth from a bull into a human, it may be possible to enhance athletic performance. There are several ethical and safety concerns associated with gene doping, which has led many sporting bodies to ban its use in competitions [57,58,59]. However, the technology is constantly evolving, and it is important for regulatory bodies to stay up-to-date with advancements in this field to prevent its misuse.

The targets of gene doping are genes that regulate athletic performance or physical appearance. These include genes that control muscle growth, oxygen metabolism, and endurance [60,61]. Gene doping aims to introduce or modify these target genes to enhance athletic performance beyond normal limits. Assay design for gene doping experiments involves selecting appropriate genetic markers or sequences to identify the presence or absence of the modified gene [22,62]. For example, if the goal is to introduce a gene for increased muscle growth, the assay may involve identifying changes in gene expression levels of markers such as myostatin or follistatin. The assay may also involve sequencing the genome or examining the structure of the modified gene to ensure that it has been inserted correctly. Obtaining results from gene doping experiments can be challenging as it requires accurately measuring changes in the physiological or physical characteristics of an organism. This often involves complex assays and testing protocols such as muscle biopsies, oxygen uptake measurements, or physical performance testing. Results may also need to be evaluated over time to assess the durability of the gene modification. It is important to note that gene doping is currently banned by most sports organizations due to safety and ethical concerns. While advancements in gene editing and delivery technologies may provide potential benefits in the future, they must be overseen by regulatory bodies and used responsibly to ensure the safety and welfare of athletes [21,63].

Gene doping is considered a violation of anti-doping regulations in the sports industry, and its use to enhance athletic performance carries serious legal consequences. In China, the regulations against gene doping in sports are formulated and enforced by the General Administration of Sport and related sports management agencies, with support from national laws. According to regulations governing sports event supervision, athletes or their teams found to have used gene doping techniques will face a series of penalties, including but not limited to suspension, revocation of honors received, and fines. In addition, if the actions are deemed to constitute a criminal offense, they may also face arrest, prosecution, and sentencing accordingly [61]. Overall, gene doping is unethical and anti-sports behavior that carries severe legal consequences and has irreversible impacts on those who engage in it. As such, it should be eliminated to maintain fair competition and preserve the spirit of sportsmanship [13,64].

Generally, gene doping detection methods can be divided into two categories: direct detection methods and indirect methods. The direct detection method is the same as the conventional drug doping detection method and is based on direct detections of prohibited substances or their metabolites in physiological samples. The indirect detection methods aim to measure or monitor the body responses caused by the delivery and expression of the transgenes or gene doping agents. When the detected gene doping is identical to endogenous expression products or certain substances that can be rapidly metabolized, the indirect detection method may be a good choice. However, several factors such as age, gender, ethnic background, and even natural viral infection-induced immune responses may complicate the interpretation of the results or increase the false-positive risk [65]. Therefore, direct methods are still a popular choice for developing various effective gene doping detection methods. In this section, we divide this type of method into two categories: PCR-based methods and PCR-free methods, and briefly introduce the latest research progress on their application for gene doping detection.

### 3.1. Typical PCR Methods

Unlike the inherent gene, gene doping normally uses cDNA; thus, their exon/exon junctions can be unique target regions to distinguish them from the relevant intrinsic genes [62]. As the gold standard of gene detection, PCR detection technology has been widely studied because of its high accuracy and reproducibility [22,23]. Not surprisingly, it has been widely used in the field of gene doping detection in recent years. WADA also issued a special guideline on the application of PCR technology in gene doping detection. One of the items in the guideline recommends the use of whole blood as the sample from which DNA is extracted, and that can be used as a template for transgene detection using quantitative PCR with target-specific primers and hydrolysis probes [66]. To date, several quantitative PCR-based gene doping detection technologies have been reported for use in gene doping control analysis. Such methods include the use of several starting materials (e.g., whole blood, plasma, and urine) and different PCR systems (e.g., real-time PCR and digital PCR) [19,22,62,67].

**Figure 7:**
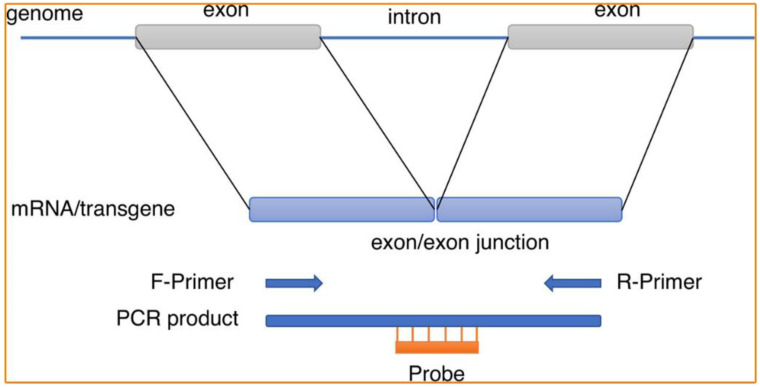
Probe and primer design for quantitative PCR detection of a transgene in a gene-doping control. (Reprinted with permission from [19] Copyright 2021, American Chemical Society).

For example, Ryder’s group successfully detected gene doping in horses with the help of quantitative real-time PCR (qPCR) [68]. Two approaches, including the ligation of sequence-ready adapters to qPCR products and qPCR assays using tailed primers, were applied to provide direct analysis of the amplified qPCR products from five candidate genes by next-generation sequencing without adopting additional amplification techniques. Tozaki et al. reported their study on the robustness, e.g., the specificity and sensitivity, of digital PCR and real-time PCR in transgene detection. Based on the use of substituted primers and probes that matched or incompletely matched the target template, this study realized low-copy transgene detection by using nested digital PCR [19] (Figure 7). The development of these methods has resulted in the gradual development of PCR technology into an accurate and credible method to detect gene doping. However, none of these methods have been implemented in accredited anti-doping laboratories due to a lack of validation [65]. Furthermore, most of those protocols involve multiple steps, which increase the risk of cross-contamination and require considerable skill, time, and expensive equipment facilities [69]. Therefore, new approaches involving simpler and faster sample manipulation would be interesting alternatives to these developed protocols. It is worth mentioning that PCR methods established using SYBR fluorescent dyes as signal probes are not applicable when addressing the detection of gene doping in real samples. The reason is that this method uses a fluorescent dye that is non-specific in recognizing double-stranded DNA, does not recognize specific double-strands, and is highly susceptible to false positive results when analyzing biological samples.

The future development direction of PCR-based methods in doping detection can be summarized as follows: (1) Improved Sensitivity: PCR methods will continue to evolve to enhance their sensitivity in order to detect extremely low levels of doping agents. This will involve the development of more efficient amplification techniques and advanced detection technologies. (2) Multiplexing Capabilities: There is a growing demand for the simultaneous detection of multiple doping agents in a single analysis. Future developments will focus on enhancing the multiplexing capabilities of PCR methods, enabling the detection of several target genes or molecules in a single reaction. (3) Rapid Testing: Efforts will be made to reduce the turnaround time required for doping detection using PCR methods. This involves the optimization of protocols, the simplification of sample preparation steps, and the introduction of faster amplification and detection technologies. (4) Biomarker Discovery: Research will continue to identify new and specific biomarkers that can serve as indicators of doping. PCR methods will play a crucial role in amplifying and detecting these biomarkers, thereby facilitating their validation and incorporation into doping detection protocols. (5) Non-Invasive Sampling: Non-invasive sampling methods, such as saliva or urine testing, are gaining popularity in doping control. The development of PCR-based methods that can amplify and detect doping biomarkers from non-invasive samples will be an area of focus for future development. (6) Integration with Other Technologies: PCR methods will be integrated with other advanced technologies, such as microfluidics, nanotechnology, and high-throughput sequencing, to further enhance the performance and capabilities of doping detection. This integration will enable improved sensitivity, increased throughput, and reduced sample volume requirements. (7) Standardization and Quality Assurance: The standardization of PCR-based doping detection methods is essential for ensuring reliable and consistent results. Efforts will be made to establish international standards, guidelines, and quality assurance programs to ensure the accuracy and reproducibility of PCR-based doping tests. These future developments in PCR methods aim to enhance the effectiveness, efficiency, and reliability of doping detection, ultimately contributing to maintaining fair play and integrity in sports.

### 3.2. Sequencing-Based Methods

In contrast to conventional PCR methods, loop-mediated isothermal amplification (LAMP) is a novel method that can amplify nucleic acids with high specificity, efficiency, and rapidity under isothermal conditions. The LAMP technique offers several advantages over traditional PCR-based methods, including simplified reaction conditions and a faster amplification process. Typically, LAMP is carried out at a constant temperature (60–65 °C) and can achieve 10^9^-fold amplification within 1 h [70]. While the logistics of LAMP are much simpler than those of PCR, the amplification principle is more complex. A key to the underlying amplification scheme is the use of primers that generate foldback structures and their subsequent extensions by a strand-displacing DNA polymerase [65,69,70]. With the help of DNA polymerase amplification, the cascade amplification of the target gene sequence can be completed, and the detection of gene doping can be realized. Its simplicity, high specificity, and robustness make it an attractive option for nucleic acid detection in resource-limited settings. For example, Leuenberger et al. reported their utilization of LAMP as an alternative to PCR in the development of a novel approach to detect gene doping. Based on the proper design of primers to trigger the LAMP reaction with high specificity and efficiency, this method can be used for the simple, rapid, and selective detection of gene doping with the naked eye [65]. Other than LAMP and PCR-based methods that target one exon/exon sequence in the intron-less transgenes, next generation sequencing (NGS)-based assays offer higher throughput and cover greater numbers of potential doping areas in gene doping [71,72]. It allows for the rapid and simultaneous sequencing of millions of DNA fragments, providing detailed information about the genetic makeup of an individual or a sample. In gene detection, NGS plays a crucial role in identifying, characterizing, and understanding the genetic variations associated with various diseases and conditions. It offers a comprehensive view of the entire genome, capturing both coding and non-coding regions, which helps in identifying disease-causing mutations, structural variants, and gene expression patterns.

In addition, whole-genome resequencing (WGR), a technique used in genomics to obtain the complete DNA sequence of an individual’s genome. It involves comparing the individual’s DNA sequence to a reference genome to identify differences, including single nucleotide polymorphisms (SNPs), insertions, deletions, and structural variations [73]. NGS and WGR are both powerful techniques used in genomics research to analyze DNA sequences. There are some differences between them. NGS refers to a set of high-throughput sequencing technologies that enable the parallel sequencing of millions of DNA fragments. It involves the fragmentation of the DNA sample into smaller pieces, the attachment of adapters to these fragments, and their amplification through multiple cycles of sequencing. WGR refers to the process of sequencing the entire genome of an organism, including both coding and non-coding regions, to identify genetic variations in comparison to a reference genome. In WGR, the DNA of an individual or a population is sequenced de novo or compared to a known reference genome. The advantages of WGR for SNP analysis mainly include: (1) comprehensive coverage: WGR allows for an extensive analysis of the entire genome, providing a comprehensive view of all potential SNPs present. (2) Discovery of rare and novel variants: By sequencing the entire genome, WGR enables the identification of rare and novel SNPs that may not be captured using other methods [73]. And the advantages of NGS for SNP analysis mainly include: (1) high-throughput: NGS enables the simultaneous analysis of millions to billions of DNA fragments in a single sequencing run. (2) Accuracy: the use of various error correction algorithms during data processing helps to minimize sequencing errors, resulting in more reliable SNP calling [71,72].

It is worth noting that different DNA enzymes can also be used to detect gene doping and could be used to replace the complicated PCR detection method [65]. Recent advancements in DNA enzyme technology offer a promising alternative for detecting gene doping. DNA enzymes, also known as DNAzymes or catalytic DNA, are synthetic single-stranded DNA molecules with enzymatic activity. For example, polymerases [74], which can catalyze the synthesis of polymers, specifically nucleic acids such as DNA or RNA. They are widely used in PCR-based detection and DNA sequencing (such as sanger sequencing and next-generation sequencing (NGS)). These techniques employ DNA polymerase to incorporate modified nucleotides, which terminate the DNA synthesis reaction at specific positions. By analyzing the termination pattern, the sequence of the original DNA, which can cleave the phosphodiester bonds within a DNA or RNA molecule can be determined as endonucleases [75]. They are widely used in various applications involving DNA and RNA analysis and detection. They can recognize specific DNA sequences and cut the DNA at or near these sequences. This allows for the analysis of DNA fragments of specific sizes and facilitates the isolation of genes or specific DNA regions of interest. exonucleases [76], which can catalyze the degradation of nucleic acids by removing nucleotides from the ends of DNA or RNA molecules. They can recognize and cleave specific sequences of DNA or RNA, making them essential tools in molecular biology research and analysis. metal ion-dependent DNAzymes [77], which are artificial DNA enzymes that exhibit catalytic activity in the presence of specific metal ions. These DNA molecules can fold into specific three-dimensional structures, allowing them to bind metal ions and perform catalytic reactions. By designing DNAzymes that require specific metal ions for their catalytic activity, it is possible to develop assays that detect the presence of target gene doping. G-quadruplex-based catalytic nucleic acids [78], which are DNA or RNA molecules that possess both a G-quadruplex structure and catalytic activity. Their unique structure and catalytic activity make them valuable tools for sensitive, specific, and versatile genetic analysis and gene expression regulation.

Overall, these DNA enzymes can recognize specific target sequences within the genetic material and catalyze specific reactions. The advantage of using DNA enzymes for gene doping detection lies in their ability to directly amplify and detect the presence of modified genes without the need for PCR. This eliminates the complexity and time required for PCR amplification and simplifies the overall detection process. Additionally, DNA enzymes can provide high specificity and sensitivity, allowing for accurate identification of gene doping. Moreover, DNA enzymes are more stable and cost-effective compared to traditional PCR-based methods. They do not require sophisticated laboratory equipment or specialized techniques for analysis. This makes them more accessible and feasible for widespread use in anti-doping efforts. In conclusion, the use of different DNA enzymes for gene doping detection shows great potential for replacing the complicated PCR detection method. These enzymes offer advantages such as simplicity, accuracy, high specificity, and cost-effectiveness. Further research and development in this field can contribute to the enhancement of anti-doping measures in the sporting community.

### 3.3. CRISPR Methods

**Figure 8:**
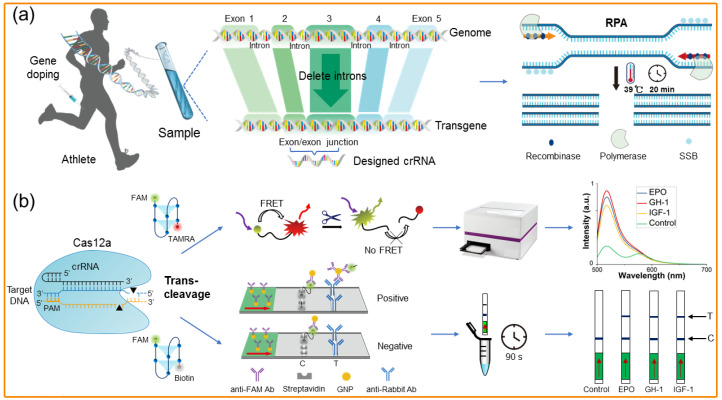
Working principle of the CRISPR/CasGDP. (**a**) Sample preparation for gene doping detection. Injected gene doping targets contain exon/exon junctions that are used to design the Cas12a-crRNAs. Transgenes are amplified by recombinase polymerase amplification. (**b**) Fluorescence or lateral flow device -based GD detection by CRISPR/Cas12a. (Reprinted with permission from [82] Copyright 2022, American Chemical Society).

In addition to the commonly used DNAzymes, the CRISPR/Cas (Clustered Regularly Interspaced Short Palindromic Repeats) technique, which is well-known for its gene-editing function, has also exhibited great promise in the biosensing and bioassay fields in recent years [57,79,80,81]. Due to the DNase/RNase properties of the CRISPR/Cas system, its combination with various nucleic acid recognition and amplification reactions can be used for the versatile construction of a large variety of powerful methods to provide highly efficient detections of many target analytes, including gene doping. Compared to the traditional DNAase-based assay established for qPCR, LAMP, and NGS, the CRISPR detection method offers improved sensitivity, speed, specificity, and versatility in detecting specific DNA sequences. CRISPR is a widely used technology in genome editing and genetic engineering. It is a natural immune system found in bacteria and archaea that can locate and cut DNA, allowing for the repair, replacement, or deletion of specific gene sequences. The core principles of CRISPR technology include three main components: single guide RNA (sgRNA), CRISPR RNA (crRNA), and a fluorescence reporter gene. (1) sgRNA is synthesized from two components: a guiding RNA (tracrRNA) and a specific sequence. The guiding RNA portion binds to the protein Cas, helping the Cas enzyme accurately locate the target DNA. The specific sequence is used to recognize the target DNA. It pairs with a specific sequence on the target DNA, forming a “signal sequence” that allows the Cas enzyme to accurately identify and bind to the target DNA. (2) crRNA is an RNA molecule that is synthetically designed in advance to specifically pair with a specific sequence in the target DNA. In CRISPR genome editing, crRNA is artificially designed to precisely guide the activity of the Cas enzyme, enabling it to accurately cleave the target DNA sequence. (3) Fluorescent reporter genes are commonly used as labeling methods in CRISPR technology. By combining a fluorescent gene with the target gene, it is possible to observe whether the target gene has been successfully modified. Recently, our group successfully developed a novel CRISPR/Cas-based method to evaluate gene doping. Due to CRISPR/Cas12a and multiplexed recombinase polymerase amplification (RPA, a nucleic acid amplification technique that allows for the rapid and specific amplification of DNA or RNA from a sample using recombinase enzymes to facilitate the formation of DNA helices between target DNA sequences and primers), this method showed high specificity and sensitivity for rapid, robust, and on-site gene doping detection. Furthermore, it successfully detected transgenes in a cell model and combined a four-plexed microfluidic chip to simultaneously detect the three transgenes [82] (Figure 8). Additionally, Sung’s group successfully developed an in vitro CRISPR-Cas9 cleavage system to analyze the site-specific exogenous gene doping of human erythropoietin [69]. The outstanding superiority of CRISPR-Cas9 capabilities has enabled direct, simple, selective, and sensitive assays when using the method. Apart from these, CRISPR-deadCas9 has also been reported to be used for genetic doping detection. Sung’s Group successfully completed the detection of exogenous human erythropoietin gene doping based on CRISPR/deadCas9. Under optimal conditions, the developed assay successfully achieves highly sensitive detection of gene doping in a whole blood sample at concentrations of 12.3 fM (7.41 × 10^5^ copies) and up to 10 nM (6.07 × 10^11^ copies) within 1 h [57]. However, to our knowledge, neither CRISPR-Cas13 nor CRISPR-Cas13a have been reported for use in gene doping detection. This may be due to the fact that CRISPR-Cas13 and CRISPR-Cas13a are primarily applied to shear against RNA.

### 3.4. MS-Based Methods

In addition to the above nucleic acid-based detection methods, the traditional drug doping detection methods based on MS have also been successfully applied to gene doping detection. Recently, Thevis’s group developed a series of MS-based gene doping detection methods and successfully applied the traditional analytical doping detection methods to detect new dopants [20,83,84]. For example, they used HPLC-HRMS/MS to identify the presence of the exogenous protein Cas9 from the bacterium *Streptococcus pyogenes* in athlete serum samples. By monitoring the misuse of the CRISPR/Cas system by athletes, the presence of gene doping in athletes can be assessed. Last but not least, the biosensors, especially various aptasensors that are traditionally used for drug doping detection, can also be used for gene doping detection [13,64,85]. In summary, compared with traditional PCR-based gene doping detection methods, PCR-free gene doping detection methods have unique advantages in terms of operability, detection cost, and equipment requirements. In the author’s opinion, in a future development trend, PCR-free gene doping detection methods will hopefully gradually replace PCR methods and become the main method to detect gene doping.

The future development direction of PCR-free methods for detecting doping is as follows: (1) Improving Sensitivity: Enhancing the sensitivity of PCR-free methods is crucial for detecting doping substances at even lower concentrations. This can be achieved through advanced sample preparation techniques, optimized detection platforms, and innovative signal amplification strategies. (2) Multiplexing Capabilities: Developing PCR-free methods that are capable of simultaneous detection of multiple doping substances will greatly enhance their efficiency and practicality. This can be achieved by designing specific probes or primers targeting different doping targets and utilizing advanced multiplexing detection technologies. (3) Portable and Point-of-Care Devices: The development of portable and point-of-care devices for PCR-free doping detection would enable rapid and on-site analysis. These devices should be easy to use, user-friendly, and capable of providing accurate and reliable results in a short period of time. (4) Integration with Other Analytical Techniques: Integrating PCR-free methods with other analytical techniques, such as MS or immunoassays, can improve the selectivity and accuracy of doping detection. This integration can provide complementary information and enhance the overall detection capabilities. (5) Standardization and Validation: To ensure the widespread adoption of PCR-free methods for doping detection, it is necessary to establish standard protocols and validation procedures. This includes defining performance criteria, optimizing sample preparation methods, and conducting extensive validation experiments using reference materials and blind samples. (6) Data Analysis and Interpretation: Developing advanced data analysis and interpretation algorithms specifically designed for PCR-free doping detection will be crucial. This will enable accurate and reliable identification of doping substances based on the specific signals generated by the detection method. Overall, the future development of PCR-free methods for detecting doping will focus on improving sensitivity, enabling multiplexing capabilities, developing portable devices, integrating with other analytical techniques, standardizing protocols, and enhancing data analysis and interpretation algorithms.

## 4. Comparison of the Reported Assays

Doping detection assays can be broadly classified into two categories: drug doping detection and gene doping detection. Here are comparisons of drug doping detection and gene doping detection (Table 2) and commonly used doping detection assays (Table 3). The most commonly used for doping detection are GC-MS and LC-MS/MS, which are based on MS. These are powerful analytical techniques that allow for the detection of trace amounts of banned substances and low-level metabolites of banned substances in biological samples. Other methods, such as fluorescence, electrochemical, and colorimetric methods, have advantages over MS-based methods in terms of ease of detection and cost, but the accuracy and sensitivity of the results are not as good as those of MS. For the detection of gene doping, there are currently two main types of assays: PCR and PCR-free methods. Both methods have their own advantages and disadvantages. The PCR detection method has better sensitivity and can detect even a single copy of target DNA. Additionally, the PCR method has superior specificity due to the use of two primers that bind specifically to the target sequence. In contrast, PCR-free has advantages in terms of time spent on detection, technology, and instrumentation requirements. In summary, all of these assays have their own advantages and limitations, and each one is used based on specific requirements and circumstances. The choice of assay depends on the substances being tested and the accuracy, sensitivity, and specificity required for detection.

## 5. Conclusions and Outlook

Over an extended period in previous years, the utilization of doping has profoundly undermined the principles of fairness and justice in competitive sports and has had a significant impact on the holistic development of sports. Fortunately, stringent measures taken by regulatory agencies against doping, coupled with continuous advancements and maturation of doping detection technologies, have effectively mitigated the illicit use of prohibited substances among athletes. This review emphasizes the latest advancements in detection methodologies for doping control analysis, encompassing organic drug doping and gene doping, over the past decade. Noteworthy progress in the field of MS has facilitated the identification of minute quantities of banned substances or their metabolites within biological samples, thereby laying the groundwork for the development of auxiliary technologies such as fluorescence methods, electrochemical methods, colorimetric methods, and diverse biosensors. Furthermore, a comparative analysis of the merits and drawbacks of each detection method is conducted. Finally, a succinct discussion on the prospective directions for detecting these two forms of doping is provided.

Regarding the future trajectory of doping detection, the author posits that it primarily encompasses the following three facets: (1) Advancement of high-throughput detection sensors to enable simultaneous identification of multiple categories of doping substances; (2) integration of nucleic acid amplification, nanomaterial amplification, and other novel signal amplification technologies to achieve highly sensitive detection of doping molecules within complex samples; (3) construction of integrated and miniaturized sensors to cater to the requirements of on-site detection of doping substances. It is crucial to note that the development of prohibited substances capable of enhancing athletic performance is an ongoing endeavor aimed at evading monitoring. Consequently, the pursuit of doping detection will persist, necessitating continuous updates and innovations in doping detection methodologies.

## Figures and Tables

**Table 1:** Prohibited substances in WADA and available detection methods.

Category	Representative Substance	Available Detection Methods
S0 Non-approved substances		GC-MS/MS; LC-MS/MS; HILIC-HRMS
S1 Anabolic agents	Anabolic androgenic steroids (AAS)	GC-MS/MS; LC-MS/MS; GC-C/IRMS; LC–IM–Q/TOF; LC–HRMS/MS; GC–HRMS/MS; LC-Ag^+^CIS/MS/MS
S2 Peptide hormones, growth factors, related substances, and mimetics	Erythropoietin (EPO); Growth hormone (GH)	LC-MS/MS; ELISA; Transcriptomics; Proteomics; SAGE; SELDI-TOF MS; LC-MS/MS; LC-HRMS/MS; Immunoassay
S3 Beta-2 agonists	Salmeterol; Tretoquinol	LC-MS/MS; UHPLC-HRMS; LC-HRMS/MS
S4 Hormone and metabolic modulators	Aromatase inhibitors	GC-MS/MS; GC-C/IRMS; LC-MS/MS; Hyperpolarized NMR based metabolomics
S5 Diuretics and masking agents	Desmopressin; Probenecid; Acetazolamide	GC-MS/MS; LC-MS/MS
S6 Stimulants	Cocaine; Strychnine	GC-MS/MS; LC-MS/MS; ESI-MS/MS; LC-HRMS/MS;
S7 Narcotics	Morphine; Pentazocine	LC-MS/MS
S8 Cannabinoids	Cannabinoids	GC-MS/MS; LC-MS/MS
S9 Glucocorticoids	Cortisone; Dexamethasone	LC-MS/MS
M1 Manipulation of blood and blood components	Blood doping	LC-MS/MS; Proteomics; Transcriptomics
M2 Chemical and physical manipulation	Sample substitution and/or adulteration	Vigilance
M3 Gene and cell doping	Gene editing; Gene silencing; Gene transfer technologies	Polymerase chain reaction (PCR) (WADA-approved); NGS; WGR; HPLC-MS; CRISPR-Cas based systems
P1 Beta-blockers	Bunolol; Propranolol	LC-MS/MS

**Table 2:** Comparison of drug doping detection and gene doping detection.

Categories	Drug Doping Detection	Gene Doping Detection
Definition	The use of prohibited drugs to enhance performance in sports	The use of gene therapy or genetic manipulation to enhance athletic performance
Detection Methods	Testing urine or blood samples for the presence of banned substances.	Analyzing DNA samples to detect specific genetic modifications or enhancements.
Types of Enhancements Detected	Use of stimulants, anabolic steroids, peptide hormones, etc.	Introduction of specific genes to improve muscle growth, oxygen utilization, endurance, etc.
Detection Window	Limited timeframe after drug administration, as drugs are metabolized and excreted from the body.	Potential for indefinite detection as genetic modifications can persist for a longer period.
Challenges	Constant development of new undetectable substances.	Complex and evolving methods of gene delivery and manipulation.
Ethical Concerns	Public health risks and long-term detrimental effects on athletes’ health.	Alteration of natural genetic traits, fairness in competition, and potential health risks.

**Table 3:** Comparison of the reported assays for doping detection.

Method	Detection Time	Detection Cost	Target	Sample Information	LOD	LOQ	Ref.
MS based	Less than 15 min	High	pharmaceuticals active compounds	Fish sampling points	5–50 ng/g	2.0 ng/g	[33] (2020)
Fluorescence	Less than 15 min	Low	amphetamine-type stimulants	2 mL saliva	10^−3^–10^−9^ M	0.72 µM	[43] (2020)
Electroanalytical	Less than 5 min	Low	3,4-Methylenedioxyamphetamine and 3,4-methylenedioxymethamphetamine	10 μL urine	0.05–7.5 μM and 0.1–7.5 μM	37 nM and 54 nM	[50] (2022)
Colorimetric	Less than 10 min	Low	amphetamine-type stimulants	20 μL urine	0–50 μg/mL	0.66 μg/mL	[38] (2022)
Biosensors	More than 120 min	Medium	methamphetamine	saliva, serum and urine,	0.02–20 µM	20 nM	[16] (2022)
PCR-based	More than 120 min	High	myostatin gene	2.2 μL horse plasmid solution	No mention	No mention	[19] (2021)
PCR-free based	Less than 40 min	Medium	human EPO gene	10 μL human plasmid solution	10^−11^–10^−8^ M	1 aM	[82] (2023)

## Data Availability

Data available on request due to restrictions, e.g., privacy or ethical.
